# Prognostic Factors in a Large Nationwide Cohort of Histologically Confirmed Primary and Secondary Angiosarcomas

**DOI:** 10.3390/cancers11111780

**Published:** 2019-11-12

**Authors:** Marije E. Weidema, Uta E. Flucke, Winette T.A. van der Graaf, Vincent K.Y. Ho, Melissa H.S. Hillebrandt-Roeffen, Dutch Nationwide Network and Registry of Histo- and Cytopathology (PALGA)-Group, Yvonne M.H. Versleijen-Jonkers, Olga Husson, Ingrid M.E. Desar

**Affiliations:** 1 Department of Medical Oncology, Radboud University Medical Center, 6525 GA Nijmegen, The Netherlands; Marije.Weidema@radboudumc.nl (M.E.W.);; 2 Department of Pathology, Radboud University Medical Center, 6525 GA Nijmegen, The Netherlands; Uta.Flucke@radboudumc.nl; 3 Department of Medical Oncology, Netherlands Cancer Institute, 1066 CX Amsterdam, The Netherlands; w.vd.graaf@nki.nl; 4 Royal Marsden NHS Foundation Trust, Sarcoma Unit, London SW3 6JJ, UK; 5 Netherlands Comprehensive Cancer Organization (IKNL), 3511 DT Utrecht, The Netherlands; V.Ho@iknl.nl; 6 Institute of Cancer Research, Division of Clinical Studies, Sutton SM2 5NG, UK; 7 Division of Psychosocial Oncology and Epidemiology, Netherlands Cancer Institute, 1066 CX Amsterdam, The Netherlands

**Keywords:** angiosarcoma, pathology review, epidemiology, prognostic factors, clinical subtype

## Abstract

Angiosarcoma (AS) is a rare sarcoma of endothelial origin, arising spontaneously (primary AS) or after external damage such as radiation therapy or UV exposure (secondary AS). To date, reliable assessment of prognostic factors has proven difficult, due to disease rarity and heterogeneity of study cohorts. Although large registries provide relatively large AS patient series, these cases often lack histological confirmation. This study aimed to analyze AS prognostic factors in a large nationwide cohort of histologically confirmed cases, established through linkage of clinical data from the Netherlands Cancer Registry and pathology data from the Dutch pathology registry (PALGA). All cases were reviewed by an expert pathologist, showing a 16% discordance rate. Multivariable Cox regression survival analysis among 479 confirmed AS patients revealed remarkably poorer overall survival (OS) for primary AS compared to secondary AS (7 vs 21 months, Hazard ratio (HR) = 1.5; 95% confidence interval (CI) = 1.2–1.9). Age above 65 years, male gender, and no surgical treatment also significantly correlated to worse OS. Overall, OS was relatively poor, with a median of 13 months (95% CI = 10–16 months) and 22% five-year survival rate. With this study, we illustrate AS heterogeneity in clinical behavior and show for the first time better survival for secondary AS compared to primary AS.

## 1. Introduction

Angiosarcoma (AS) is a rare vasoformative sarcoma, with a reported incidence of about 1.5/1,000,000 persons per year [1]. AS can present everywhere in the body, either as a sporadic tumor or as a secondary tumor related to external damaging factors such as radiation therapy (RTx), chronic lymphedema or UV exposure. Therefore, heterogeneity within AS is considerable and associations between clinical presentation and prognosis remain uncertain.

Survival for AS patients is generally poor with reported five-year survival rates of around 40% [2,3,4], dropping to 15% in metastatic patients [2]. There are contradictory findings concerning outcomes for clinical subtypes, as some studies reported either favorable or worse prognosis for RTx induced or soft tissue (ST) AS [4,5,6,7], whilst others did not find prognostic value for clinical subtypes [2,8]. It could be speculated that primary (sporadic) AS has a different mechanism of development than secondary AS. Whereas primary AS supposedly originates from mesenchymal stem cells or progenitor cells and can therefore occur anywhere in the body, secondary AS develops due to external damage and is exclusively localized at the damaged site. However, the difference in clinical behavior between primary and secondary AS is not yet fully known.

As with other rare cancers, limited availability of reliable data is one of the main barriers for conducting AS research. The available literature mainly consists of retrospective series and uses different AS classifications. Only few studies accrued sufficient patient numbers to perform multivariable analyses.

The systematic collection of patient data in (inter)national registries may help overcome this barrier. However, potential pitfalls of using such databases lie in missing data and in the reliability of pathology data. In sarcomas, central pathology review revealed a 14–42% discordance rate [9,10]. In the case of AS, diagnosis provides an additional challenge, as histological features are heterogeneous and can vary both within one tumor as well as between cases [11].

So far, the largest AS cohort studies retrieved clinical data of 391 to 1250 AS patients from the SEER database (Surveillance, Epidemiology, and End Results Program, USA) [12,13,14,15] or National Cancer Database (USA), respectively [16,17]. In the Netherlands, both nationwide clinical data (Netherlands Cancer Registry; NCR) and pathology reports (Dutch Nationwide Network and Registry of Histo- and Cytopathology; PALGA) are available for research purposes. Pathology and clinical data of a large number of AS cases are available in both registries and can be linked. Using this linkage, we aimed to describe a large Dutch cohort of histologically confirmed AS cases to study patient demographics, tumor characteristics and outcome. Multivariable survival analysis revealed for the first time better survival for secondary AS patients compared to primary AS patients. Additional prognostic factors included age, gender, and surgery.

## 2. Results

### 2.1. Pathology Review

A total of 1125 cases with reported AS (*n* = 1010) or epithelioid hemangioendothelioma (EHE) (*n* = 115) diagnosis were available in the Netherlands Cancer registry, of which 656 cases were available for review. Additional stainings were performed on 155 samples. Out of 559 reported AS cases, 467 (84%) were histologically confirmed (Supplementary Table S1). Twelve additional AS cases were identified among cases with a different or uncertain diagnosis, yielding 479 confirmed AS cases.

### 2.2. Patient Characteristics

Median age at diagnosis was 70 years (range 2—98) and the majority of patients were female (65%; 312/479) (Table 1). Most patients in our cohort had secondary AS (54%; 259/479), compared to 35% primary AS (169/479). For 51 patients (11%), tumor localization and etiology were unknown. Median age was significantly higher in secondary AS patients, 74 years compared to 66 and 62 years for primary and unknown AS, respectively (*p* < 0.01) (Table 1). Secondary AS patients more often presented with superficial tumors (*p* < 0.01). Radiation-induced (RT) AS was the largest clinical subtype represented (34%; 163/479) (Table 2). Median time since radiation therapy was 8 (range, 1–19) years and the majority (88%; 144/163) were localized in the breast area, whereas 4/163 (2%) were localized in an organ and the remaining 15 cases had different other localizations (9%). Most of the UV associated (UV) AS cases were localized on the scalp (55/79; 70%), followed by the face (23/79; 29%) and neck (1/79; 1%). Almost all of the post lymphedema (Stewart Treves) cases (16/17; 94%) were located in an extremity and one case presented in the breast area. The majority of patients (64%; 306/479) received surgery, whereas adjuvant radiotherapy was administered in 49 cases (10%). Use of (neo) adjuvant chemotherapy was reported in only 3% of patients (12/479). Patients with unknown AS more often presented with distant metastasis at time of diagnosis (*p* < 0.01), received less surgery (*p* < 0.01) and were most likely to receive primary chemotherapy (*p* < 0.01) (Table 1).

### 2.3. Survival

Median overall survival (OS) was 12.8 months for the entire cohort (95% confidence interval (CI) 10.1–15.5 months) (Table 2). Overall one- and two-year survival rates were 52.6% (95% CI 48.1–57.1%) and 35.7% (95% CI 31.4–40.0%) respectively. The five-year OS rate was 21.9% (95% CI 18.2–25.6%), with a 10-year OS of 9.4% (95% CI 5.9–12.9%). Patients with primary breast AS had the longest median OS (32.5 months, 95% CI 0–116.0 months, Table 3), whereas patients with visceral AS had a median OS of only 2.2 months (95% CI 1.1–3.3 months). There was no significant difference in survival between the different subtypes of secondary AS (RT, UV or Stewart Treves; *p* = 0.260). Secondary AS patients had significantly better median OS (20.6 months, 95% CI 16.1–25.1) than primary (7.2 months, 95% CI 5.1–9.3) and unknown AS (3.7 months, 95% CI 0–8.8) (*p* < 0.01).

### 2.4. Prognostic Factors

**Figure 1:**
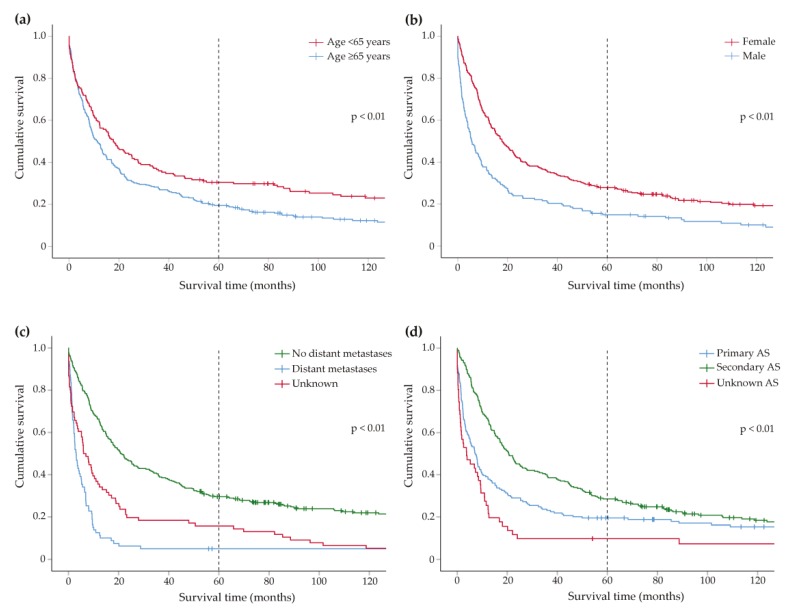
Overall survival. **(a)** Kaplan–Meier curves showing the difference in 10-year overall survival between patients <65 years and ≥65 years, **(b)** Kaplan–Meier curves showing the difference in 10-year overall survival between male and female patients, **(c)** Kaplan–Meier curves showing the difference in 10-year overall survival between patients with and without distant metastasis, **(d)** Kaplan–Meier curves showing the difference in 10-year overall survival between clinical subgroups.* Dotted line represents five-year overall survival.

Factors univariably associated with poor OS were age ≥65 years, male gender, primary AS, deep seated tumors, and distant metastases (all *p* < 0.01) (Table 3, Figure 1). Surgery as well as (neo)adjuvant radiation therapy both correlated with better OS (*p* < 0.01). In multivariable analysis, primary AS patients had significantly poorer survival than secondary AS patients (HR 1.51; 95% CI 1.20–1.89) (Table 4). Furthermore, age, gender, tumor depth, presence of distant metastases, and surgery were identified as independent predictors.

## 3. Discussion

The aim of this study was to analyze prognostic factors in primary and secondary AS (after RT, UV, and chronic lymphedema). By using national databases of linked pathological and clinical data, we established the largest histologically confirmed AS cohort to date, containing almost 500 cases.

Overall, survival in our cohort was relatively poor with a five-year OS rate of 22%. Secondary AS patients had a significantly better survival than primary AS patients. This is in contrast with most previous larger studies (*n* > 100), in which OS did not correlate with previous radiation therapy in multivariable analysis [2,3,16]. Although primary breast AS patients in our cohort appeared to have a relatively favorable survival (median OS 33 months, 95% CI 0–116 months), the large confidence interval indicated a large variance between patients. Of the two previous studies that did describe a correlation between clinical origin and survival, Fayette et al. reported a worse OS for primary liver AS (relative risk 12.6), but only 7 (4%) liver AS patients were included in their study, thus strongly limiting the reliability of this finding [4]. Furthermore, AS localization in an area of pre-existing chronic lymphedema was previously reported to predict poor OS (HR 2.0; 95% CI 1.1–3.6) [18].

We found that older age was a negative prognostic factor. This was previously observed in two other series of UV associated head and neck AS patients (HR 1.04; 95% CI 1.02–1.05) [12] and patients with localized AS (HR 2.1; 95% CI 1.7–2.5), respectively [16].

As expected, distant metastases at diagnosis were predictive of worse OS in our cohort. Two previous studies reported a similar negative correlation with OS (RR 2.5 in all AS and HR 2.97; 95% CI 1.7–5.3 in head and neck AS, respectively) [4,12]. Of note, in the third study metastatic disease only predicted disease-free survival and not OS [3], which may be explained by the relatively short follow-up of median 13 months. Treatment with surgery correlated with better OS in our study and was reported in only one previous cohort of localized AS patients (HR 0.37; 95% CI 0.16–0.87) [18].

Female patients had significantly better survival in our study. Gender has not been previously reported as a prognostic factor, although gender distribution highly varied between studies (31–84% female patients) [12,18]. Tumor depth was also found to predict OS in our study. It was only investigated in one previous study, in which it had no prognostic value [16]. However, AS tumor depth can be difficult to measure, depending on the localization of the tumor, thus limiting its use as a clinical indicator. In contrast to our findings, tumor size was a consistent predictor of OS in several studies [2,12,16,18]. This could be explained by the fact that tumor size was missing in 86% of cases in our study. Overall, reported prognostic factors vary between studies, probably due to differences between cohorts and clinical factors included in the analyses.

These differences between cohorts are also reflected in reported median OS. Median OS in our cohort was about 13 months, compared to 39–43 months in most of the larger retrospective studies in localized AS [2,16,18]. Our study did include 17% patients with metastatic disease, which was only the case in two previous studies (16–19% distant metastases) [3,4]. Median OS in these cohorts was 18 and 42 months, respectively, with a higher 5-year OS rate (41–43%) than in our cohort. This is most likely due to the composition of the patient cohorts, although the study of Wang et al. did not describe characteristics of the 113 analyzed patients and can therefore not be compared to our study [3]. Clinical characteristics in the study of Fayette et al. slightly differed on significant prognostic factors compared to our cohort [4], with for instance less patients with visceral AS (9% vs 14%) and more patients receiving surgery (75% vs 63%). As our cohort exclusively consists of patients with an AS confirmed diagnosis, our survival rates may provide a more accurate estimation of OS and reflect the aggressive nature of AS.

When interpreting previous results, it is important to consider that the AS cases in most of the large registries were not reviewed. Given the considerable discordance rate of 14–42% sarcoma diagnoses [9,10], these databases probably also contain patients with other diagnoses, consequently confounding the outcomes. In our study, we were able to establish a large cohort of histologically confirmed AS. Some previous studies also performed pathology review but contained no more than 204 patients [2,3,4,18]. One of these studies reported a discordance rate of only 3% (7/204) [4], compared to 16% in our cohort. Their low rate may be explained by the fact that all cases were derived from expert sarcoma centers and by the implementation of expert pathology review in the French clinical sarcoma practice [19,20]. The higher discordance rate in our cohort most likely reflects the challenges regarding AS diagnosis [21] and emphasizes the great value of pathology review in retrospective registry-based AS studies.

Another factor to take into account by interpretation of prognostic factors is the proportion of missing data in the databases. In our study, we used the Netherlands Cancer Registry to establish our database and had to accept that clinical characteristics such as tumor size and tumor depth were missing in 67–85% of cases. This is at least partly explained by the natural behavior of AS, for instance cutaneous AS often presents as difficult to measure, diffuse, multifocal lesions. We could not find factors related to missing values in our study and therefore we assumed the data to be missing at random (ignorable). We incorporated the cases with missing data in our multivariable model, though it has also been shown that complete case analyses provide less precise estimates compared to analyses where imputation techniques are used [22]. Missing data are quite common in AS studies (Supplementary Table S2). So far, only Sinnamon et al. reported inclusion of missing data in multivariable analysis [16]. Four out of the seven other studies did not provide any details regarding missing data [2,3,12,18]. Altogether, transparency on missing data allows to assess reliability of the outcomes and to compare different studies. Future studies should explore the nature of missing data in more depth. By striving to establish not only complete but also uniform registries [23], the reliability of analyses with these data can be further improved.

Besides missing data, there are a few other limitations of our study which are inherent to the retrospective nature of our database analysis. The NCR does not document disease specific survival or the specific types of systemic treatments received. Since the NCR only records information concerning the initial diagnosis, we were unable to investigate tumor recurrence. We did have data regarding previous malignancies and their treatments, which enabled the identification of radiation induced AS. However, data regarding radiation therapy for benign conditions or before 1990 could be missing. This was partly overcome by the clinical information we retrieved from the pathology reports.

Our results illustrate AS heterogeneity in terms of patient characteristics and clinical behavior. It is important to take this heterogeneity into account while designing therapeutic intervention studies, to improve patient selection and therefore increase the chance of success. For future epidemiological studies, our findings stress the importance of pathology review and transparent reporting on missing data. These steps are necessary to improve data quality, thereby enabling adequate comparison between different AS studies and ultimately paving the way to a better understanding of this rare cancer. Another promising possibility for future studies would be to apply deep learning strategies using digital clinical, radiological, and/or pathological images obtained at time of diagnosis to enable identification of novel prognostic features within these images.

## 4. Materials and Methods

### 4.1. Clinical and Pathology Data

We initiated our study by a nationwide query on AS cases diagnosed between 1989 and 2014 using the Netherlands Cancer Registry (NCR). We also included Epithelioid Hemangioendothelioma (EHE), since these can be difficult to distinguish from AS. Patients were identified using the ICD-O-3 histology codes for angiosarcoma (M9120), lymphangiosarcoma (M9170), EHE (M9133), and hemangioendothelioma (M9130). The NCR compiles data of all individuals newly diagnosed with cancer in the Netherlands and provided patient and tumor characteristics and information on primary treatment, collected from hospital records by dedicated data managers. Follow-up information on vital status was obtained through linkage with the Municipal Personal Records Database, updated until 31-01-2019. To retrieve the original specimens and pathology reports, the patients from the NCR were linked to the PALGA database [24] through a joint registration number.

### 4.2. Pathology Review

On the basis of the data provided by PALGA, we selected tumor samples and pathology reports for revision. Of the available cases, Hematoxylin and Eosin (H&E) slides and, if present, immunohistochemistry stainings were reviewed by an expert sarcoma pathologist (UF) and researcher (MW). Additional immunohistochemistry stainings (CD31, ERG, CAMTA1, TFE3, HHV8, SMA) were performed on selected cases. After revision, confirmed AS cases were selected for further analysis and categorized on the basis of etiology. Cases were classified as radiation therapy induced (RT), on the basis of data from the NCR, and complemented with clinical information from the pathology reports. All cutaneous AS from the head and neck region were classified as UV induced (UV). Those cases in which the pathology report mentioned chronic lymphedema in the area of the tumor were considered post lymphedema AS (Stewart Treves syndrome).

### 4.3. Statistical Analysis

For descriptive and survival analyses, RT, UV, and Stewart Treves cases were classified as secondary AS, whereas all other sporadic cases were classified as primary AS. Cases with unknown anatomical site were considered unknown AS. Differences in patient and clinical characteristics were examined using analysis of variance (ANOVA) for continuous variables and χ2-tests for categorical variables, where appropriate. Overall survival (OS) and survival rates (1-, 2-, 5- and 10-year OS) were calculated using the Kaplan–Meier method and life tables. Differences in OS were determined using the log-rank test.

To identify prognostic factors for survival, univariable analyses were performed using Cox proportional hazards modeling. On the basis of clinical relevance and significance in univariable tests (*p* < 0.05), factors were selected for multivariable Cox regression analysis. We excluded tumor grade from our analyses, as this is no longer considered applicable to AS diagnosis [25]. Missing data were classified as ‘unknown’ and included in the analyses, to be able to include all patients. All statistical analyses were performed with IBM SPSS Statistics (Armonk, NY, USA), version 25.0.0.1.

### 4.4. Approval

Ethical approval was obtained from the local certified Medical Ethics Committee of the Radboud University Medical Center, Nijmegen, The Netherlands (File number 2016-2686), Supervisory Committee of the NCR (File number K16.195) and scientific board and PALGA Privacy committee (LZV 2017-57).

## 5. Conclusions

In conclusion, we report on prognostic factors in a cohort of almost 500 histologically confirmed angiosarcoma cases. We show for the first time that patients with secondary AS have a significantly better OS than primary AS patients. In addition, multivariable analysis revealed female gender, age below 65 years, and non-metastatic disease to be favorable prognostic factors. Our findings illustrate AS heterogeneity and its overall poor prognosis. AS heterogeneity warrants further study and should be taken into account in the design of future AS patient studies to improve patient selection and increase the chance of finding better treatment options for these rare cancer patients.

## Figures and Tables

**Table 1:** Patient demographics and clinical characteristics.

Variable	All Patients N = 479 No. (%)	Primary AS N = 169 No. (%)	Secondary AS N = 259 No. (%)	Unknown AS N = 51 No. (%)	*p*-value
**Age**					<0.01^1,2^
Median age (range)	70 (2–98)	66 (2–96)	74 (38–98)	62 (15–83)	
**Gender**					<0.01
Male	167 (34.9)	74 (43.8)	62 (23.9)	31 (61.8)	
Female	312 (65.1)	95 (56.2)	197 (76.1)	20 (39.2)	
**Time since radiation therapy**					
Median time (years, range)	8 (1–19)	-	8 (1–19)	-	-
**Tumor size**					0.601
≤ 5 cm	43 (9.0)	14 (8.3)	25 (9.7)	4 (7.8)	
> 5 cm	26 (5.4)	7 (4.1)	14 (5.4)	5 (9.8)	
Unknown	410 (85.6)	148 (87.6)	220 (84.9)	42 (82.4)	
**Tumor depth**					<0.01
Superficial	134 (28.0)	21 (12.4)	112 (43.2)	1 (2.0)	
Deep	25 (5.2)	16 (9.5)	3 (1.2)	6 (11.8)	
Unknown	320 (66.8)	132 (78.1)	144 (55.6)	44 (86.3)	
**Distant metastasis**					<0.01
No	324 (67.6)	106 (62.7)	201 (77.6)	17 (33.3)	
Yes	79 (16.5)	37 (21.9)	19 (7.3)	23 (45.1)	
Unknown	76 (15.9)	26 (15.4)	39 (15.1)	11 (21.6)	
**Surgery**					<0.01
Yes	306 (63.9)	107 (63.3)	192 (74.1)	7 (13.7)	
No	173 (36.1)	62 (36.7)	67 (25.9)	44 (86.3)	
**Residual disease**					0.273
R0	12 (2.5)	4 (3.7)	7 (3.6)	1 (14.3)	
R1	4 (0.8)	0 (0.0)	4 (2.1)	0 (0.0)	
Unknown	463 (96.7)	103 (96.3)	181 (94.3)	6 (85.7)	
**Radiation therapy**					0.217
Neoadjuvant	3 (0.6)	0 (0.0)	2 (0.8)	1 (2.0)	
Adjuvant	49 (10.2)	20 (11.8)	27 (10.4)	2 (3.9)	
Neoadjuvant + adjuvant	2 (0.4)	0 (0.0)	2 (0.8)	0 (0.0)	
Primary	41 (8.6)	9 (5.3)	26 (10.0)	6 (11.8)	
No	384 (80.2)	140 (82.8)	202 (78.0)	42 (82.4)	
**Chemotherapy**					<0.01
Neoadjuvant	5 (1.0)	2 (1.2)	3 (1.2)	0 (0.0)	
Adjuvant	7 (1.5)	6 (3.6)	1 (0.4)	0 (0.0)	
Primary	47 (9.8)	13 (7.7)	22 (8.5)	12 (23.5)	
No	420 (87.7)	148 (87.6)	233 (90.0)	39 (76.5)	

^1^ primary vs secondary AS, ^2^ unknown vs secondary AS.

**Table 2:** Overall survival per clinical subtype.

Clinical Subtype	No. (% of Total)	Median OS (95% CI) in Months	*p*-value
All	478 (100)	12.8 (10.1–15.5)	
**Primary AS**	**169 (35)**	**7.2 (5.1–9.3)**	
Visceral	67 (14)	2.2 (1.1–3.3)	0.000
Primary breast	28 (6)	32.5 (0.0–116.0)	
Skin (non UV associated)	27 (6)	15.1 (9.3–21.0)	
Deep soft tissue	25 (5)	13.1 (3.5–22.8)	
Heart and vessels	12 (3)	2.8 (1.4–4.2)	
Other	10 (2)	6.9 (0.0–14.3)	
**Secondary AS**	**259 (54)**	**20.6 (16.1–25.1)**	
Radiation associated	163 (34)	22.8 (17.3–28.2)	0.260
Skin (UV associated)	79 (17)	18.7 (10.3–27.1)	
Stewart Treves	17 (4)	17.8 (7.5–28.1)	
**Unknown AS**	**51 (11)**	**3.7 (0.0–8.8)**	NA

OS = overall survival, CI = confidence interval.

**Table 3:** Survival analysis: clinical characteristics and overall survival.

Variable		Overall Survival	
	No. (%)	Median OS (95% CI)^1^	*p*-value^2^
**Age**			<0.01^3^
<65 years	167 (35)	17.8 (12.4–23.2)	
≥65 years	312 (65)	11.3 (8.9–13.7)	
**Gender**			<0.01^3^
Female	312 (65)	17.7 (13.8–21.7)	
Male	167 (35)	5.6 (3.7–7.6)	
**Clinical subtype**			<0.01^3^
Secondary AS†	259 (54)	20.6 (16.0–25.1)	
Primary AS	169 (35)	7.2 (5.1–9.4)	
Unknown	51 (11)	3.7 (0.0–8.8)	
**Tumor size**			0.65
≤ 5 cm	43 (9)	16.8 (8.7–25.0)	
> 5 cm	26 (5)	13.1 (2.3–23.9)	
Unknown	410 (86)	12.6 (10.0–15.2)	
**Tumor depth**			<0.01^3^
Superficial	134 (28)	32.1 (14.5–49.9)	
Deep	25 (5)	2.8 (0.3–5.3)	
Unknown	320 (67)	9.6 (7.5–11.8)	
**Distant metastases**			<0.01^3^
No	324 (68)	20.8 (16.3–25.3)	
Yes	79 (17)	3.1 (2.0–4.1)	
Unknown	76 (16)	6.0 (3.3–8.6)	
**Surgery**			<0.01^3^
Yes	306 (64)	23.2 (16.4–30.1)	
No	173 (36)	4.0 (2.1–6.0)	
**Radiation therapy**			<0.01^3^
(Neo)adjuvant	54 (11)	26.1 (9.6–42.5)	
Primary	41 (9)	9.2 (5.4–13.1)	
No	384 (80)	12.6 (9.6–15.6)	
**Chemotherapy**			0.28
(Neo)adjuvant	12 (3)	13.7 (0.0–27.8)	
Primary	47 (10)	10.3 (6.5–14.2)	
No	420 (88)	12.8 (9.7–16.0)	

OS = overall survival, CI = confidence interval. †= RT, UV and Stewart Treves AS, ^1^ in months, ^2^ log-rank test, ^3^ *p* < 0.05.

**Table 4:** Multivariable survival analysis.

Variable		Overall Survival	
	No. (%)	Hazard Ratio (95% CI)	*p*-value
**Age**			<0.01^2^
<65 years	167 (35)	Ref.	
≥65 years	312 (65)	1.89 (1.52 – 2.36)	
**Gender**			0.02^2^
Female	312 (65)	Ref.	
Male	167 (35)	1.31 (1.05 – 1.64)	
**Clinical subtype**			
Secondary AS^1^	259 (54)	Ref.	
Primary AS	169 (35)	1.51 (1.20 – 1.89)	<0.01^2^
Unknown	51 (11)	1.04 (0.71 – 1.52)	0.84
**Tumor depth**			
Superficial	134 (28)	Ref.	
Deep	25 (5)	1.93 (1.18 – 3.16)	<0.01^2^
Unknown	320 (67)	1.15 (0.89 – 1.48)	0.29
**Distant metastases**			
No	324 (68)	Ref.	
Yes	79 (17)	2.09 (1.54 – 2.85)	<0.01^2^
Unknown	76 (16)	1.72 (1.31 – 2.25)	<0.01^2^
**Surgery**			<0.01^2^
Yes	306 (64)	Ref.	
No	173 (36)	2.52 (1.93 – 3.29)	
**Radiation therapy**			0.10
(Neo)adjuvant	54 (11)	Ref.	
Primary	41 (9)	0.91 (0.55 – 1.51)	
No	384 (80)	1.26 (0.89 – 1.78)	

CI = confidence interval, ^1^ RT, UV and Stewart Treves AS, ^2^ *p* < 0.05.

## Supplementary Materials

The following are available online at https://www.mdpi.com/2072-6694/11/11/1780/s1, Table S1: Reported and definite diagnoses after pathology review, Table S2: Missing data in retrospective angiosarcoma cohort studies with multivariable analyses (n ≥ 100 patients).
